# Relationships among Musical Aptitude, Digit Ratio and Testosterone in Men and Women

**DOI:** 10.1371/journal.pone.0057637

**Published:** 2013-03-08

**Authors:** Jeremy C. Borniger, Adeel Chaudhry, Michael P. Muehlenbein

**Affiliations:** Department of Anthropology, Indiana University, Bloomington, Indiana, United States of America; University of Washington, United States of America

## Abstract

Circulating adult testosterone levels, digit ratio (length of the second finger relative to the fourth finger), and directional asymmetry in digit ratio are considered sexually dimorphic traits in humans. These have been related to spatial abilities in men and women, and because similar brain structures appear to be involved in both spatial and musical abilities, neuroendocrine function may be related to musical as well as spatial cognition. To evaluate relationships among testosterone and musical ability in men and women, saliva samples were collected, testosterone concentrations assessed, and digit ratios calculated using standardized protocols in a sample of university students (N = 61), including both music and non-music majors. Results of Spearman correlations suggest that digit ratio and testosterone levels are statistically related to musical aptitude and performance *only* within the female sample: A) those females with greater self-reported history of exposure to music (p = 0.016) and instrument proficiency (p = 0.040) scored higher on the Advanced Measures of Music Audiation test, B) those females with higher left hand digit ratio (and perhaps lower fetal testosterone levels) were more highly ranked (p = 0.007) in the orchestra, C) female music students exhibited a trend (p = 0.082) towards higher testosterone levels compared to female non-music students, and D) female music students with higher rank in the orchestra/band had higher testosterone levels (p = 0.003) than lower ranked students. None of these relationships were significant in the male sample, although a lack of statistical power may be one cause. The effects of testosterone are likely a small part of a poorly understood system of biological and environmental stimuli that contribute to musical aptitude. Hormones may play some role in modulating the phenotype of musical ability, and this may be the case for females more so than males.

## Introduction

Claude-Levi Strauss proclaimed “Since music is the only language with the contradictory attributes of being at once intelligible and untranslatable, the musical creator is a being comparable to the gods, and music itself the supreme mystery of the science of man” [1]. It has been suggested that human musical ability, like bird song, may represent a trait evolved through the process of sexual selection, specifically in response to mate choice [2]. Music takes time to learn and master, time that could be spent searching for food or other activities. Music may attract unwanted attention from predators and competitors, but may also demonstrate desired abilities to members of the opposite gender. Could musical ability have been, or continue to be, considered a trait which demonstrates survivability, intelligence, parental ability, “good genes,” or any other factors deemed important for mate choice? It is possible that there should be some sexual dimorphism in musical ability, and that this could be functionally linked to various biological factors.

Music affects a variety of psychological and physiological systems, influencing heart function and mental state [3]. The neuroendocrine system acts as a central coordinator of a number of these functions, including cognition. Hormones are related to spatial abilities in both men [4], [5] and women [6], [7] in complex ways. For example, elevated early androgen exposure due to congenital adrenal hyperplasia is associated with masculinized spatial abilities in females [8], but several studies have identified no relationships between male-biased spatial performance and adult circulating androgens [9] or digit ratios, a putative proxy for early androgens [8]. Spatial abilities are intimately related to learning music [10], [11], listening to music [12], [13], and musical aptitude [14]–[16]. By logical connection, hormones may be related to musical ability in interesting ways. Furthermore, high testosterone phenotypes are often thought to be indicative of mate quality as well, as men with good energetic availability and low parasite/pathogen load can afford to maintain higher levels of testosterone [17]. But how might testosterone be related to general musical capability, preference and exposure? And more importantly, why might this be so?

Using a sample of young adults, including both music and non-music majors, amateur and professional musicians, we *hypothesized* (A) that testosterone would be directly associated with musical ability in both genders, using a validated measure of musical audiation (perception of rhythm and tone), and other measures including orchestra/band chair position. We also *hypothesized* (B) that testosterone would be higher in music than in non-music students.

Because digit ratio (2D:4D) has been considered by some to be a proxy measure of fetal androgen exposure [18], and has been demonstrated as at least a crude direct measure in both humans [19] and mice [20], we *hypothesized* (C) that those with greater musical ability would have a lower digit ratio, supposedly indicative of higher prenatal androgen exposure. Androgen concentrations and digit ratios are related to mate quality and preference in several vertebrate species [21]. A key knowledge gap exists in associating musical ability as an honest/costly signal of mate quality. Tentative evidence for this may be gathered by identifying correlations between musical aptitude and phenotypic signals such as androgen concentrations and digit ratios. For example, Slumming and Manning [22] found lower left hand digit ratios in men that ranked higher in an orchestral hierarchy (i.e., presumably better professional musicians have exposure to higher levels of testosterone in utero). Fetal brain organization is mediated, in part, by androgens [23]; therefore these hormones may have significant effects on future musical aptitude. Of course, these hormones may also be influenced through organization effects during puberty [24] as well as activational effects later in life [25].

Directional asymmetry can be calculated from the difference between the right and left hand digit ratios [26], [27]. According to Benderlioglu and Nelson [26], positive values of directional asymmetry are indicative of a male-typical pattern of a lower digit ratio in the right hand relative to the left hand. Because of this, we also *hypothesized* (D) that males who score higher in musical aptitude would have lower directional asymmetry, while females who score higher in musical aptitude would have greater directional asymmetry.

## Methods

Participant recruitment and sampling took place on the Indiana University, Bloomington campus between November 2010 and April 2011. Participants were recruited in person, as well as using email listserv announcements and flyers. Ethical approval was obtained from the Indiana University Human Subject’s Committee (study #1010002333). Written informed consent was obtained from all subjects prior to participating.

Exclusion criteria included: any known infectious disease or endocrine, metabolic, reproductive or immunosuppressive disorder; history of drug or alcohol abuse or malnutrition; weight change greater than ten pounds in the past year; history of chronic disorders (e.g., chronic obstructive pulmonary disease, congestive heart failure, renal or kidney failure, etc.); history of depression or other psychiatric disorder; currently taking any prescription or nonprescription drugs; history of intracranial disease or head trauma; and recent history (past three months) of surgery or other injury.

All women were not pregnant and reported having consistent, regular menstrual cycles (mean length of 27.07 days, standard deviation of 2.943 days). In the absence of estradiol and progesterone levels, it is not possible for us to accurately determine the point in the menstrual cycle at which each female participated, which is certainly a limitation of the present study. 29.4% of the female participants reported being on hormonal birth control. Although others have reported elevations in testosterone associated with hormonal birth control [28], which would otherwise confound the results of the present study, we identified no statistical relationships (independent t-tests) between birth control status and testosterone (p = 0.939), AMMA (p = 0.11) and self-reported musical aptitude (p = 0.359). Therefore all female participants are categorized into a single group rather than by birth control status in subsequent analyses.

Each participant completed the Advanced Measures of Music Audiation (AMMA) test (GIA Publications, Inc.). The test has thirty questions preceded by three practice questions where the answer is revealed and directions are given. The entire test takes approximately twenty minutes to complete. Answers are recorded on a scantron sheet, while musical statements are heard through stereo headphones. The test taker is played a short piano segment (∼five seconds), followed by a pause (∼two seconds), and then another short piano segment (∼five seconds). The second piano segment is either different tonally [t], different rhythmically [r], or the same as the initial segment [s]. There is only one correct response for each question. The participant was able to adjust the volume of the test, but was not able to skip ahead, pause, or go back. AMMA reliability scores are 0.84 for tonal, 0.85 for rhythm, and 0.88 for total score [29]. The AMMA has been used extensively to predict the music performance achievement of university students [29]. AMMA answer sheets were scored by hand using scoring masks (test overlays allowing for quick scoring by hand) and scoring directions provided by GIA Publications, Inc. This allows for raw, raw adjusted, standard, and percentile rank scores to be assessed for rhythm, tonal, and total scores (rhythm+tonal). In order to compare groups effectively, only raw scores were used in statistical analyses.

For each participant, marks were made in the center of the proximal crease (where the palm of the hand meets the fingers) of the second (2D) and fourth (4D) digits on both hands. Photocopies were made of both hands, and the lengths of the second and fourth digits were determined to the nearest 0.1 millimeter using digital calipers following the protocol of Manning [18]. Digit ratios for each hand were determined in addition to directional asymmetry (difference in right hand 2D:4D from left hand 2D:4D). All measurements were made by a single investigator to eliminate inter-observer error.

Participants completed a detailed questionnaire regarding lifetime musical exposure, which included items on the amount of formal and informal training, music listening experiences, parental-induced exposure to music, among others. A score of “self-reported musical exposure” (SRME) was calculated by summing the results of the following questions (scored using a 0 to 4 likert-type scale of “never {0} to “all of the time” {4}): I listen to music; I listen to a wide variety of music; I perform music; I sing; I attend live musical events; I listen to music to relieve stress; and I perform music to relieve stress.

Participants were provided with saliva collection materials. Participants donated saliva samples immediately upon waking (prior to eating, drinking, teeth brushing or any other activities) on three consecutive days. Labeled tubes were stored in participants’ home freezers prior to transfer to the laboratory where they were stored at −80°C prior to analyses. Samples for individual participants were pooled by transferring exactly one milliliter from each of the participant’s three samples into a new single tube, and vortexing for five minutes. Because hormone levels can fluctuate significantly within a person from day to day, we contend that using a pool of three samples consecutive represents a better baseline hormone level than can be determined using just a single sample. Samples collected on multiple days are often used to determine basal hormone levels [30], and these samples can be pooled to yield an average among the samples/days [31].

Testosterone levels were determined using enzyme immunoassay kits from Salimetrics, LLC according to the manufacturer’s instructions (expanded range salivary testosterone EIA kit #1–2402). The sensitivity of the assay was <1.0 pg/ml. The correlation coefficients for each of the curves were better than 0.99. High- and low-level controls were included in each standard curve (2 plates), and results for the controls in the assay were within established confidence limits (16.666±1.737 pg/ml and 13.85±1.61 pg/ml for the low controls; 212.678±1.436 pg/ml and 199.654±5.499 pg/ml for the high controls). Intra-assay coefficient of variation was assessed using the mean coefficients of variation of sample duplicates. Intra-assay coefficient of variation was 2.81% for testosterone. Inter-assay coefficient of variation was assessed using the mean coefficients of variation of high and low control duplicates in two separate assays. Inter-assay coefficient of variation was 11.01% for testosterone.

Statistical analyses were conducted using PASW Statistics 18 (IBM SPSS). Testosterone values were log transformed to correct for departure from normal distribution, as is usually the case with hormone samples. Level of significance was set at 0.05. Spearman correlations were used to measure relationships between variables within gender and music status (music versus non-music students). Mann-Whitney U tests were used to measure differences between groups.

Adjustments for multiplicity were not applied. However, the statistical analyses did employ a gatekeeping strategy for which the primary four hypotheses were evaluated in order listed above. Results of all secondary analyses (beyond the first null hypothesis that was not rejected) cannot be considered confirmatory. Given the number of statistical tests conducted, it is possible that results with alpha between 0.05 and 0.005 are not statistically meaningful. Post-hoc power analyses were conducted using G*Power (Version 3.1.3, Heinrich-Heine University, Dusseldorf, Germany) for all statistical tests that yielded results with p>0.05.

## Results

61 subjects (27 male; 34 female) completed all aspects of the project. 21 of them were music majors (undergraduate or graduate; 12 male, 9 female). Average age of participants was 21 years (range 18–29). 12 had broken a finger or had damaged their digits in some way, and were therefore excluded from the digit ratio analyses.

### Basic Gender Differences

Males had significantly higher testosterone levels than females (male mean: 182.63 pg/ml; female mean: 80.69 pg/ml; p<0.001) (see Table 1). Males had significantly lower digit ratios than females in both the left (male 2D:4D mean: 0.951; female 2D:4D mean: 0.971; p = 0.007) and right (male 2D:4D mean: 0.938; female 2D:4D mean: 0.970; p<.001) hands. Males and females did not score significantly differently on the total AMMA raw score (male mean: 59.81; female mean: 56.53; p = 0.125).

**Table 1:** Differences in mean variables in relation to gender and musical status.

Variable	Male Mean(N = 27)	Female Mean (N = 34)	*P*
2D:4D Right Hand*	0.938	0.97	**<0.001**
2D:4D Left Hand*	0.951	0.971	**0.007**
2D:4D Directional Asymmetry*	−0.013392	−0.001040	0.215
Testosterone	182.64 pg/ml	80.69 pg/ml	**<0.001**
AMMA Total Raw Score#	59.81	56.53	0.125
Self-Reported Musical Exposure	18.33	17.74	0.738
**Variable**	**Male Music Student Mean** **(N = 12)**	**Male Non-Music Student Mean (N = 15)**	***P***
2D:4D Right Hand*	0.936	0.94	0.909
2D:4D Left Hand*	0.961	0.943	0.239
2D:4D Directional Asymmetry*	−0.0251	−0.00384	0.184
Testosterone	179.08 pg/ml	185.48 pg/ml	0.961
AMMA Total Raw Score#	63.83	56.6	**0.018**
Self-Reported Musical Exposure	21.75	15.6	**0.007**
**Variable**	**Female Music Student Mean (N = 9)**	**Female Non-Music Student Mean (N = 25)**	***P***
2D:4D Right Hand*	0.968	0.971	0.814
2D:4D Left Hand*	0.977	0.968	0.814
2D:4D Directional Asymmetry*	−0.0094	0.003	0.370
Testosterone	97.42 pg/ml	74.67 pg/ml	0.082
AMMA Total Raw Score#	62.2	54.48	**0.010**
Self-Reported Musical Exposure	23.1	15.8	**0.001**

* Sample size is different for digit ratio measures (20 males, 29 females) because some participants had history of damaged digits.

# AMMA = Advanced Measures of Music Audiation.

### Musical Ability and Experience

**Figure 1:**
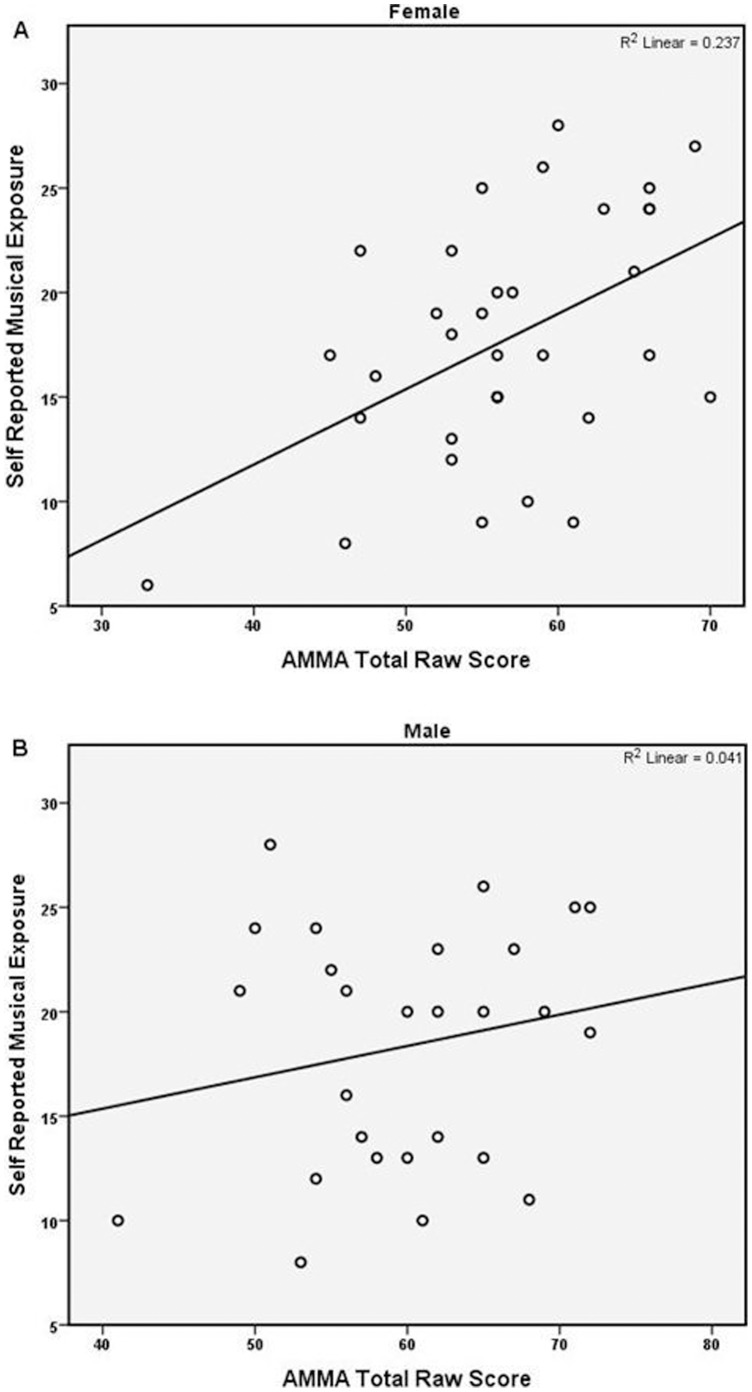
Self-reported musical exposure compared to Advanced Measures of Music Audiation (AMMA) total raw score (Females N = 34; Males N = 27).

The resultant score of the “self-reported musical exposure” SRME questionnaire (minimum = 0, maximum = 28) was significantly positively correlated with the total raw score on the AMMA for the females (R = .410, p = 0.016) but not the males (R = 0.136, p = 0.500), and not in the total sample when genders were combined (see Tables 2, 3 and 4, and Figure 1).

**Table 2:** Spearman correlation coefficients for several variable of interest across the whole sample (males and females combined). (N = 49 for 2D:4D measures; N = 61 for all other measures).

Variable	Testosterone (log pg/ml)	AMMA Total Raw Score#	Left Hand 2D:4D	Right Hand 2D:4D	DirectionalAsymmetry of 2D:4D	Self-Reported Musical Exposure
Testosterone (log pg/ml)	1	.159	−.317*	−.437**	−.173	.097
AMMA Total Raw Score#		1	.040	.120	−.017	.296*
Left Hand 2D:4D			1	.582**	−.367**	.102
Right Hand 2D:4D				1	.490**	−.194
Directional Asymmetry of 2D:4D					1	−.318*
Self-Reported Musical Exposure						1

* Correlation is significant at the 0.05 level.

** Correlation is significant at the 0.01 level.

# AMMA = Advanced Measures of Music Audiation.

**Table 3:** Spearman correlation coefficients for several variable of interest within the female sample (N = 29 for 2D:4D measures; N = 34 for all other measures).

Variable	Testosterone (log pg/ml)	AMMA Total Raw Score#	Left Hand 2D:4D	Right Hand2D:4D	Directional Asymmetry of 2D:4D	Self-Reported Musical Exposure
Testosterone (log pg/ml)	1	−.062	.081	−.239	−.224	.224
AMMA Total Raw Score#		1	.095	.075	−.132	.410*
Left Hand 2D:4D			1	.430*	−.546**	.257
Right Hand 2D:4D				1	.453*	.052
Directional Asymmetry of 2D:4D					1	−.230
Self-Reported Musical Exposure						1

* Correlation is significant at the 0.05 level.

** Correlation is significant at the 0.01 level.

# AMMA = Advanced Measures of Music Audiation.

**Table 4:** Spearman correlation coefficients for several variable of interest within the male sample (N = 20 for 2D:4D measures; N = 27 for all other measures).

Variable	Testosterone (log pg/ml)	AMMA Total Raw Score#	Left Hand 2D:4D	Right Hand 2D:4D	DirectionalAsymmetry of 2D:4D	Self-Reported Musical Exposure
Testosterone (log pg/ml)	1	.040	−.134	.072	.170	−.027
AMMA Total Raw Score#		1	−.115	.303	.206	.136
Left Hand 2D:4D			1	.435	−.392	−.040
Right Hand 2D:4D				1	.552*	−.477*
Directional Asymmetry of 2D:4D					1	−.348
Self-Reported Musical Exposure						1

* Correlation is significant at the 0.05 level.

** Correlation is significant at the 0.01 level.

# AMMA = Advanced Measures of Music Audiation.

Of those with experience playing an instrument (N = 51), participants rated their own current instrument proficiency against what they thought was the average for all others they had ever met (using a likert-type scale consisting of the following choices: high above average, above average, average, poor, and far below average). This score significantly positively correlated with total raw score on the AMMA across the whole sample (R = 0.289, p = 0.040; Table 2), but again only in females when gender was analyzed separately (females R = 0.399, p = 0.035; males R = 0.100, p = 0.651; Tables 3 and 4).

### Hormones and Music

Music students scored higher on the AMMA than non-music students. Male music student (N = 12) mean score was 63.83, male non-music student (N = 15) mean score was 56.6 (p = .018); female music student (N = 9) mean score was 62.22, female non-music student (N = 25) mean score was 54.48 (p = .010) (see Table 1).

**Figure 2:**
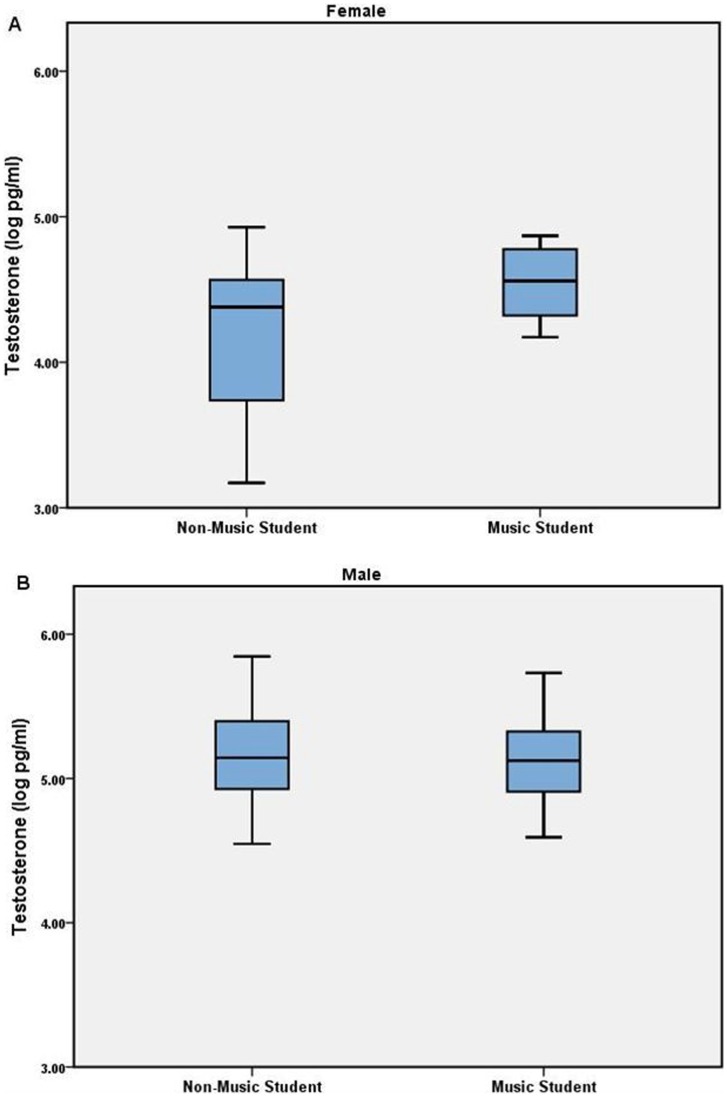
Testosterone levels in female music (N = 9) and non-music (N = 25) students, and male music (N = 12) and non-music (N = 15) students.

There was a trend towards higher testosterone levels in female music students (mean 97.42 pg/ml) compared to female non-music students (mean 74.67 pg/ml; p = 0.082) (see Figure 2 and Table 3). Given the small sample size and power of only 47% (for this comparison), this result likely reflects a false negative. There is also the remote possibility of sampling bias where female music students could have been more likely to donate samples around time of ovulation, and testosterone levels do appear to increase just prior to ovulation [32]. Review of menstrual status/history on the questionnaires did not indicate that this was the case.

For the males, there was little difference in testosterone levels between music (mean 179.07 pg/ml) and non-music (mean 185.48 pg/ml) students (p = 0.961).

If applicable, participants were asked to name their orchestra/band chair position. Within the females that reported their position (N = 9), testosterone was significantly negatively correlated with chair position (R = −0.857, p = 0.003). Therefore, lower chair position (1^st^ chair, 2^nd^ chair, etc.), and presumably greater musical ability, was associated with higher testosterone level. Within males (N = 7), no association was found between chair position and testosterone levels (R = −0.182, p = 0.696).

Self-reported musical exposure (SRME) was examined in relation to testosterone level in both sexes to identify potential links between chronic musical exposure and androgens. Among males (N = 27), testosterone (R = −0.027, p = 0.894) did not correlate with SRME. Similar results were found within the females (N = 34) (R = 0.224, p = 0.202).

There was no significant relationship between testosterone levels and scores on the AMMA within males (R = 0.04, p = 0.843) or females (R = −0.062, p = 0.729). Similar results (not shown) were identified when analyzing the AMMA tonal and rhythm scores separately.

### Digit Ratios

**Figure 3:**
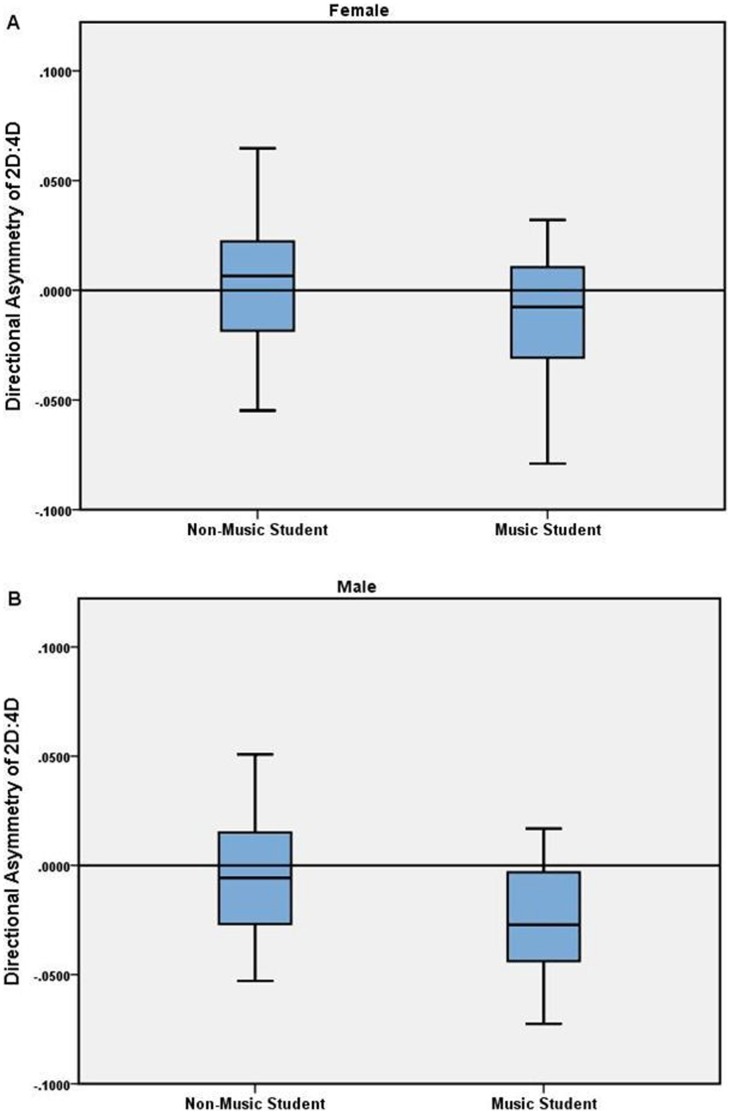
Directional asymmetry in digit ratio of female music (N = 9) and non-music (N = 20) students, and male music (N = 9) and non-music (N = 11) students.

Within the males, there were no significant differences between music and non-music students in right hand 2D:4D (p = 0.909), left hand 2D:4D (p = 0.239) or digit ratio directional asymmetry (p = 0.184). Within the females, there were also no significant differences between music and non-music students in right hand 2D:4D (p = 0.814), left hand 2D:4D (p = 0.814) or digit ratio directional asymmetry (p = 0.370) (see Figure 3). There was no significant difference between males and females in digit ratio directional asymmetry (p = 0.215). This lack of gender difference in digit ratio directional asymmetry may undermine its utility as a marker of sex hormones (including those in utero), and is therefore excluded from further analyses in the present report.

Within females that reported chair position in an orchestra/band (N = 9), left hand 2D:4D was significantly negatively correlated to chair position (R = −0.820, p = 0.007) so that those with higher left hand digit ratio (and supposedly lower fetal testosterone levels) were better musicians (top-ranked chair positions). This relationship was not significant within the male sample, possibly due in part to an extremely small sample size (N = 5).

Testosterone was not significantly correlated to right or left hand 2D:4D ratios in males or females (male right hand: R = 0.072, p = 0.762; male left hand: R = −0.134, p = 0.574; female right hand: R = −0.239, p = 0.212; female left hand: R = 0.081, p = 0.675).

## Discussion

The present study extends previous analyses on physiological and morphological correlates of musical ability by utilizing a validated measure of musical aptitude [29], [33]. As expected, music students scored higher on the AMMA than non-music students. Interestingly, history of exposure to music and self-reported instrument proficiency were positively correlated with musical aptitude (total AMMA score) in only the female, not the male sample. While such results may reflect a small sample size in our pilot project, this might also be indicative of actual gender differences in the influence of various factors. Males did not outperform females in the AMMA, but some things may be more important in influencing the development of musical aptitude in males versus females. A lack of gender differences in AMMA scores in relation to testosterone may indicate that sex steroids play a small role (if any) in influencing this measure of musical aptitude. Environmental influences (as crudely measured by the SRME questionnaire) may be more reliable in assessing gender differences in musical aptitude in relation to the AMMA. A longitudinal study from adolescence to adulthood would be necessary to adequately assess the influence of chronic music exposure throughout life.

### Digit Ratios

While digit ratio (right or left hands) did not differ significantly between music and non-music students, left hand 2D:4D did show significant relationships to orchestral/band chair position within only the female sample. That is, those females with higher left hand digit ratio (and supposedly lower fetal testosterone levels) were reportedly better musicians (more highly ranked). Slumming and Manning [22] found a significant relationship between chair position and left hand 2D:4D ratio in 14 male violinists. That study did not recruit enough females to adequately analyze within separate instrument groups. Higher ranking (low chair position) male violinists had lower left hand 2D:4D ratios than lower ranking (high chair position) violinists. The authors argue that high pre-natal androgen exposure (as revealed by low 2D:4D ratio) is an honest indicator of male fitness, and therefore musical ability may be used as a proxy measure of mate quality for females. The results of the present study indicate that the opposite relationship between left hand 2D:4D ratio and orchestral rank may exist within females. The reasons for these differences remain unclear.

While there is some evidence that prenatal testosterone levels determined via amniocentesis predict future 2D:4D ratio at age two in both males and females [19], it remains unclear whether digit ratio is an adequate proxy of circulating androgen levels in utero or adulthood [20], [26], [34]–[36].

Musicians differ from non-musicians in hand skill [37] and brain structure [38]. We neglected to collect information on handedness and so cannot control for this factor in our analyses. Whereas some research suggests that handedness is related to musical creativity [39], others suggest that handedness does not differ systematically between musicians and non-musicians [40].

### Hormones

Female music students exhibited a trend (p = 0.082) towards higher testosterone levels (mean 97.42 pg/ml) than the female non-music students (mean 74.67 pg/ml). This was not the case for males. Yet recall that those females with higher left hand digit ratio (and supposedly lower fetal testosterone levels) were reportedly better musicians (more highly ranked). This may be reflective of differences in the importance of early exposure to androgens versus adult circulating levels, which in fact may be unrelated with one another.

Within the sample of female music students, those with the most senior chair position (and presumably the most advanced musicians) had higher testosterone levels compared to other female musicians. Again, this was not the case for males. Testosterone was unrelated to total AMMA score in men and women, music and non-music students. Hassler and colleagues [41], [42] have found that testosterone levels are lower in male composers and higher in female composers, compared to non-musicians and instrumentalists. They further suggest that there may be an ‘optimum’ range of testosterone for musical creativity: at the bottom of the normal range for males, and at the top of the normal range for females. Here, we show a relationship between circulating androgen levels and musical aptitude, independent of musical creativity, in females. Perhaps it is the case that testosterone is functionally linked to *musical aptitude*, not simply creative ability, in females more so than in males.

Unfortunately, our analysis cannot account for differences in instrumental groups (e.g., 3^rd^ chair out of 10 violinists, versus 3^rd^ chair out of 3 oboes). Nor can we account for differences in amount of training or differences between vocalists versus instrumentalists in this small sample size. It is also unclear if testosterone levels are elevated in female (or all) musicians relative to non-musicians, and higher in more senior performers, as a result of competition over placement in the orchestra/band. Competition is related to social dominance and testosterone levels in human [43] and nonhuman primates [44] in complex ways. While testosterone exposure might facilitate the acquisition of musical aptitude, which may result in better chair position in an orchestra/band, the acts of continual practice and competition for chair position may itself lead to elevated testosterone levels. It must of course be noted that non-music students could have been extensively trained in music, and may of course possess superb musical abilities, despite not being music majors. Our analyses do not control for this possible covariate.

Although in our study the saliva samples were taken prior to exposure to the AMMA test, previous studies suggest that testosterone levels increase in women but decrease in men in response to listening to music [45], [46]. Fukui [45] demonstrated that this effect is strongest when each participant listens to their favorite music as opposed to other types or silence control. Although our study suggests some possible gender differences, it is unknown how chronic music exposure influences hormone levels, as previous studies have only focused on acute effects. We found no significant relationships between self-reported musical exposure (a potential measure of chronic music exposure) and hormone levels in males or females, which may indicate that no relationship exists between hormones and chronic music exposure.

Any link between music and testosterone may be mediated by their independent relationships with spatial ability. Women with higher testosterone typically score higher on measures of spatial/mathematical ability than women with lower testosterone [47]. Listening to music also increases spatial-temporal reasoning in college students [48], and musical training enhances spatial-temporal reasoning in children [10], [11]. Exposure to music during pregnancy in rats increases neurogenesis in the hippocampus and enhances spatial learning ability in subsequent offspring [49]. Listening to classical music as well as playing the piano activate brain regions important for spatial-temporal reasoning [50], [51]. Select areas of the dorsal parietal lobe have been observed to be active during both mental rotation tasks [52] and while listening to novel piano melodies [53]. Androgen receptor mRNA-containing cells have been found throughout the brain, with a heavy concentration in the vestibular nuclei, the cochlear nuclei, the medial geniculate nucleus, and the nucleus of the lateral lemniscus, suggesting that androgens may alter the central relay of auditory information [54], including music. Testosterone’s actions on brain function at the cellular level may be a mechanism by which both spatial and musical abilities are affected.

Musical aptitude is obviously a complex trait. The effects of testosterone are probably a small part of a poorly understood system of biological and environmental stimuli that contribute to musical aptitude. Parental, social, cultural and economic factors play important roles in exposure to music and musical training. It is also possible that hormones may play a role in modulating musical aptitude, and that these effects may vary by gender. Our work provides tentative support for a link between musical aptitude and androgens within females. This relationship may be dependent on competition within an orchestral setting. Lack of relationships among testosterone levels and our various measures within the male sample may be interpreted as providing no support for the costly signaling hypothesis of musical aptitude, or as a result of low statistical power in our small sample size. Additional research into the potential biological, social and environmental determinants of musical aptitude are warranted.
